# The Panel Difficulty

**Published:** 1923-12

**Authors:** 


### The Panel Difficulty.

Sir,—With regard to the differences between the medical faculty and the officials of the bureaucratically-approved societies, the following observations in season may be helpful to the seekers after truth among your readers:—

1. The specific issue: "Are you in favour of rare and refreshing fruit by forcible feeding, or are you not?" never having been put to the alleged insured person, either at a General Election or by referendum, the question of what he thinks about the minor details of the operation has not yet arisen, and the concern of the doctors, therefore, about "letting him down" is not merely premature but irrelevant.

2. The measure in dispute was imposed upon a politically impotent community in defiance alike of the Constitution, the Truck Acts, and the will of the people, under cover of a five years' lease of office obtained by false pretences in December, 1910.

3. The licence to work, alias National Insurance card, is but a device for perpetuating, under a smoke-screen of ensuring the "nation's" health, the existing class cleavage in society, or wage system, as is exemplified by the position of certain uninsured Labour officials, who have acquired their freedom through those very unions whose members they have helped the Government to enslave through the political offices of the employer nominally functioning as a party to a private industrial contract.

4. The definitely anti-social character of the happily named "scheme" of July, 1912, is shown by the fact that while well-dined Impertinence is at liberty to contract out and to "chance getting well by going to a doctor" or otherwise, at discretion, the sole alternative open to such of the fourteen millions as are burdened with opinions and a law-abiding employer is to realise the "glorious heritage" of every free-born Briton and not be long about it, the sufferings of the million or so permanently unemployed—indigent class—who could not, as a prior condition, be relieved of the price of a loaf of bread every week, owing to the failure of the machinery of the employing medium in such cases, having been derided by the Government of 1910–15 as no commercial insurance company would dream of mocking a lapsed case.

5. The sublime assurance of the Fabian busybodies and other Slave Staters who have assumed control of the Labour movement in posing as the accredited representatives of, or as having a concrete mandate from, the victims of the long-firm swindle of the national hospital, not only beggars description, but constitutes a challenge to all who subscribe to the principle of the *vox populi* as the final court of appeal. That this legislative importation from Germany has, by the way, proved serviceable in another eminently "private" matter of quarterly incidence is not without significance as indicating the ulterior motives of the original framers of the measure.

6. The "deadlock" is of interest to those for whom your correspondent speaks only in so far as the present impasse may conceivably result in putting an end to the legal brigandage by which the former's pockets are being rifled in respect to schemes for which they have no use, on which they have not been consulted, and of whose illusory benefits they have not the slightest intention of availing themselves.

Trade Unionist.

